# A Comparative XPS, UV PES, NEXAFS, and DFT Study of the Electronic Structure of the Salen Ligand in the H_2_(Salen) Molecule and the [Ni(Salen)] Complex

**DOI:** 10.3390/ijms24129868

**Published:** 2023-06-07

**Authors:** Petr M. Korusenko, Olga V. Petrova, Anatoliy A. Vereshchagin, Konstantin P. Katin, Oleg V. Levin, Sergey V. Nekipelov, Danil V. Sivkov, Victor N. Sivkov, Alexander S. Vinogradov

**Affiliations:** 1 Department of Solid State Electronics, V.A. Fock Institute of Physics, St. Petersburg State University, 7/9 Universitetskaya nab., 199034 Saint Petersburg, Russia; p.korusenko@spbu.ru (P.M.K.);; 2 Institute of Physics and Mathematics, Komi Science Centre, Ural Branch of the Russian Academy of Sciences, 167982 Syktyvkar, Russia; nekipelovsv@mail.ru (S.V.N.);; 3 Department of Electrochemistry, Institute of Chemistry, St. Petersburg State University, 7/9 Universitetskaya nab., 199034 Saint Petersburg, Russia; 4 Department of Condensed Matter Physics, National Research Nuclear University “MEPhI”, Kashirskoe Sh. 31, 115409 Moscow, Russia

**Keywords:** H_2_(Salen) molecule (C_16_H_16_N_2_O_2_), [Ni(Salen)] complex (NiO_2_N_2_C_16_H_14_), X-ray photoemission (XPS) and ultraviolet photoemission spectroscopy (UV PES), X-ray absorption fine structure (NEXAFS) spectroscopy, density-functional theory (DFT) calculations, electronic structure

## Abstract

A comparative study of the electronic structure of the salen ligand in the H_2_(Salen) molecule and the [Ni(Salen)] complex was performed using the experimental methods of XPS, UV PES, and NEXAFS spectroscopy along with DFT calculations. Significant chemical shifts of +1.0 eV (carbon), +1.9 eV (nitrogen), and −0.4 eV (oxygen) were observed in the 1s PE spectra of the salen ligand atoms when passing from a molecule to a complex, unambiguously indicating a substantial redistribution of the valence electron density between these atoms. It is proposed that the electron density transfer to the O atoms in [Ni(Salen)] occurred not only from the Ni atom, but also from the N and C atoms. This process seemed to be realized through the delocalized conjugated π-system of the phenol C 2p electronic states of the ligand molecule. The DFT calculations (total and partial DOS) for the valence band H_2_(Salen) and [Ni(Salen)] described well the spectral shape of the UV PE spectra of both compounds and confirmed their experimental identification. An analysis of the N and O 1s NEXAFS spectra clearly indicated that the atomic structure of the ethylenediamine and phenol fragments was retained upon passing from the free salen ligand to the nickel complex.

## 1. Introduction

For many years, transition metal complexes have been popular objects for studying their electron properties, as they exhibit a variety of photochemical reactions useful for solar energy conversion, photo/electrochemical catalysis, and batteries [1,2,3,4,5,6,7]. In modern materials science, interest in these complexes has expanded significantly due to the possibility of using them as monomers in the electrochemical synthesis of conductive polymers with electronic, magnetic, and catalytic properties, which are unique compared to those of their individual organic and inorganic components [8]. Electrochemical polymerization processes are based on the redox reactions between monomeric complexes in an electrolyte, which provide the covalent bonding of monomer molecules. These reactions, like photochemical ones, are associated with the electronic transitions between the occupied and vacant molecular orbitals (MOs) of a polyatomic system (molecule, complex, and molecular fragment, etc.) and lead to the formation of an electronically excited state of this system, with an electron density distribution different from that in the ground state. This, in turn, manifests itself in a change in the reactivity of the system and a number of its properties, such as ionization potential, electron affinity, and acid-base properties, as well as in a change in its geometry (bond lengths and angles, etc.) and the dipole moment.

Electrically conductive polymers synthesized from the molecular complexes [M(Schiff)] of the transition metal atom *M* with tetradentate N_2_O_2_ Schiff-base ligands from salicylaldehyde and its derivatives are currently considered as promising materials for chemical and biological sensors, electrocatalytic systems and molecular electronics systems, chemical suppliers, electrochromic devices, and supercapacitors [8,9,10]. As noted above, the efficiency of the redox processes, which are responsible for the practically important properties of such compounds, is determined by the properties of the upper occupied and lower empty MOs of these compounds, i.e., the features of their atomic–electronic structure in the ground and excited states. As a consequence of this, the properties of the [M(Schiff)] monomers, their derivatives, and their polymer layers, such as their redox activity, charge transfer (CT), polymer configuration, and atomic–electronic structure, have been repeatedly studied using various experimental techniques: ultraviolet and visible absorption [11,12,13], FTIR spectroscopy [11], EPR [11,12,14], X-ray diffraction [14], cyclic voltammetry and chronoamperometry [14,15,16], and scanning electron microscopy [16], etc. However, the results of these studies contain only indirect knowledge of the spatial localization of the MOs between which electronic transitions occur and which are thus responsible for the redox processes of these transition metal complexes. At the same time, these transitions often occur between MOs localized on different metal and ligand atoms of the complex and are thus associated with the charge transfer between them. All this significantly complicates the complete understanding and correct description of the electronic properties of these complexes, which are especially important for their practical use. Thus, for a deeper understanding of the electronic structure of monomeric complexes and the nature of their polymerization processes, detailed information about the spatial localization of the valence electronic states in these complexes is also required.

X-ray transitions of inner shell electrons differ from the valence electronic transitions probed by optical spectroscopy, because they are element-specific, atomically localized transitions. Therefore, the methods of X-ray absorption and photoelectron spectroscopy are among the most informative methods for studying the local atomic and electronic structure of various polyatomic systems (molecules, complexes, and polymers, etc.). Near-edge X-ray absorption fine structure (NEXAFS) spectra [17] are generated by dipole-allowed electron transitions from the core shells of the atoms of the system to its vacant electronic states, resulting in the formation of localized quasi-stationary X-ray excitations. The initial states of these excitations are spatially localized within the absorbing atom and are therefore described by atomic orbitals (AOs) with a certain angular symmetry. Due to the dipole nature of absorption transitions, their final states are vacant electron states with contributions from valence AOs, which are also localized in the vicinity of the absorbing atom. This spatially localized nature of the X-ray excitations of the atom in a polyatomic system makes it possible to approximate the final electron states using the one-electron MOs of a fragment (quasimolecule), which are formed by the absorbing atom and the atoms of its nearest surroundings [18].

X-ray photoelectron spectroscopy (XPS) is a widely accepted technique for probing occupied electronic states in solid, adsorbed, and gas-phase polyatomic systems [19,20,21,22]. Core-level photoemission (CL PE) spectra are used to measure the binding energy E_bind_ (BE) of the core electrons of atoms in different compounds, the energy differences (chemical shifts) of which are then analyzed to characterize the chemical state of the atoms in the compared compounds, as well as the features of the chemical bonding between these atoms. In turn, valence band photoemission spectroscopy (VB PES) is the main technique for obtaining information about the VB electronic structure. These measurements use ultraviolet (UV) and soft X-ray photons to excite UV PE and VB PE spectra, which provide information about the energy distribution of the occupied electron states in the valence band and elucidate the AO composition of the MOs responsible for these electronic states.

Compounds related to the Schiff base are very diverse in their compositions. One of the representatives of this numerous class is the H_2_(Salen) molecule. This compound, as a ligand, forms salen complexes with various metals, among which is the N,N′-ethylen-bis(salicylideneaminato) nickel(II) complex, [Ni(Salen)]. This compound is of particular interest due to the high degree of reversibility of its redox reactions (charge/discharge processes), thus causing the relatively high conductivity of the polymer [14,16,23,24,25,26,27]. It has previously been shown that combined studies based on NEXAFS, CL, and VB PES measurements are very effective in characterizing the electronic properties and chemical bonding of similar complexes of nickel porphyrins and phthalocyanine [28,29,30,31,32]. Recently, similar studies have been carried out for the molecular complex [Ni(Salen)], in order to derive information about the features of its local electronic structure [33,34]. As a result, for the [Ni(Salen)] monomer, new knowledge of the energy positions and AO composition of the valence MOs responsible for its high-lying occupied and low-lying vacant electronic states has been obtained. An analysis of the experimental data obtained for the monomeric complex [Ni(Salen)] was carried out with an emphasis on the decisive role of the square-planar NiO_2_N_2_ coordination fragment in the formation of the atomic–electronic structure of the complex. However, this approach underestimates the influence of the salen ligand on the electronic properties of the complex, which, therefore, remain not fully understood.

To refine and supplement the results obtained, in this work, we carry out a comparative X-ray spectroscopic study of the electronic structure of the salen ligand in a metal-free H_2_(Salen) molecule and the [Ni(Salen)] complex. The measured CL PE and UV PE spectra are analyzed on the basis of their density functional theory (DFT) calculations and then supplemented with an analysis of the NEXAFS spectra of the salen ligand atoms (oxygen, nitrogen, and carbon) in the H_2_(Salen) molecule and [Ni(Salen)] complex. The main goal of this study is to obtain information about the changes in the chemical state of the atoms of the salen molecule and their local electronic structure when the salen is included in the nickel complex as a ligand, as well as on the directions of the electron transfer between the complexing nickel atom and ligand atoms, using a comparative analysis of the corresponding spectra of the ligand atoms in the molecule and complex.

Finally, it should be emphasized that there are still no such combined X-ray studies of the salen complexes of transition metal atoms. This original study presents new knowledge about the features of the electronic structure of the Ni(Salen) complex, namely, the directions and magnitudes of the electron transfer between the atoms of the ligand and the complexing Ni atom; the important role of the delocalized, conjugated π system of the C 2p electron states of the salen ligand in the processes of electronic redistribution; the manifestation of electron transfer by changing the energy positions of the partial N, C, and O 2p DOS in the valence band; the strongest change in the local electronic structure of the salen ligand in the vicinity of oxygen atoms; and the presence among the unoccupied electronic states of the complex of empty π*-MOs, reflecting π-conjugation between the oxygen (nitrogen) atoms and the phenolic rings; etc. Along with our previous studies [33,34], this work provides a scientific basis for further X-ray spectroscopic studies of the local atomic–electronic structure of a large family of [M(Schiff)] complexes of transition element atoms *M*.

## 2. Results and Discussion

**Figure 1:**
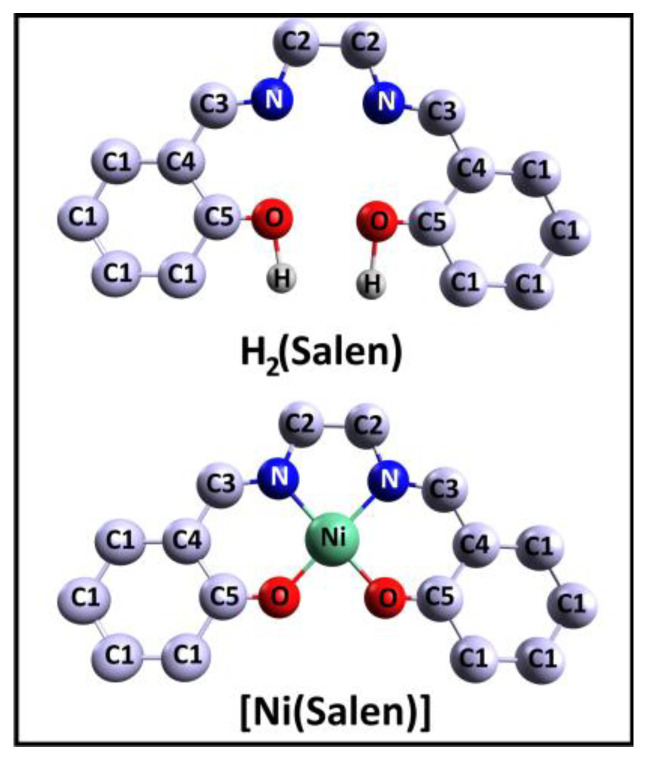
Schematic view of the salen ligand H_2_(Salen) and the [Ni(Salen)] molecular complex. Carbon atoms are divided into several numbered groups C1, C2, C3, C4, and C5 with close chemical states, as was performed in [33]. Only hydrogen atoms bonded to oxygen atoms are shown, the others are not shown.

N,N′-Ethylene-bis(salicylimine), C_16_H_16_N_2_O_2_, or H_2_(Salen), often referred to as the salen ligand, is a molecule consisting of two phenolic groups of C_6_H_5_OH, linked by an ethylenediamine bridge –N–C(H_2_)–C(H_2_)–N– (Figure 1). The structure of this molecule is characterized by the presence of several isomers that differ in the position of their two hydrogen atoms and the mutual orientation of their two benzene fragments. Based on DFT calculations, it was found that the isomer with two enol groups and a geometric configuration of C_2_ symmetry is the most energetically favorable [35]. It was also shown that the energy stabilization of this isomer is associated with the presence of two strong intramolecular hydrogen N···H bonds and that the out-of-plane distortion of the benzene fragments in all isomers is insignificant.

In turn, N,N’-Ethylene-bis(salicylaldiminato)nickel(II), C_16_H_14_N_2_O_2_Ni, or [Ni(Salen)], is a molecular complex composed of a central complexing Ni^2+^ cation and a four-dentate salen ligand (Figure 1). In the [Ni(Salen)] molecule, the central Ni atom, due to the σ-bonding, coordinates two oxygen and two nitrogen atoms of the ligand, thus forming a planar NiO_2_N_2_ coordination center (a C_2v_ point symmetry group), which is close to the square-planar one, as its interatomic distances, R(Ni–O) = 1.882 Å and R(Ni–N) = 1.889 Å, are almost equal to each other [36]. Thus, considering the structure of the [Ni(Salen)] complex, we can distinguish the following three quasi-molecular fragments within it—the NiO_2_N_2_ coordination center, as well as fragments of the salen ligand, two phenolic groups of C_6_H_5_O, and an ethylenediamine bridge. Such a consideration seems to be very constructive in a comparative analysis of the corresponding CL PE and NEXAFS spectra of the ligand from the complex and the molecule.

### 2.1. CL PES

**Figure 2:**
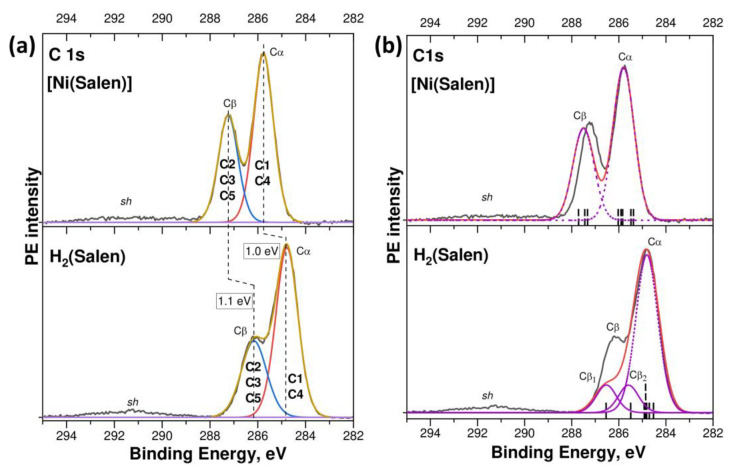
(**a**) C 1s PE spectra of H_2_(Salen) and [Ni(Salen)]: the experimental spectra measured using Al Kα radiation (*hν* = 1486.6 eV) are shown by a black solid line; the fitting components are colored in red and blue; the envelope and background are displayed by a brown and purple lines, respectively. (**b**) Comparison of the experimental C 1s PE spectra of H_2_(Salen) and [Ni(Salen)] (black curves) and the DFT calculated those (red curves); calculated energy positions of C 1s levels, shown by vertical black bars, were convoluted with a Gaussian contour with FWHM equal to 1.0 eV to facilitate the comparison with the experimental spectra; individual components of the calculated spectrum are shown by purple dashed lines.

Let us begin the discussion of the results obtained by considering the conclusions of a comparative study of the corresponding CL PE spectra of the H_2_(Salen) molecule and the [Ni(Salen)] complex. Figure 2a presents the experimental C 1s PE spectra of the H_2_(Salen) and [Ni(Salen)] on the electron BE scale, relative to the Fermi level, which are compared with those calculated using the DFT method in Figure 2b. The experimental C 1s PE spectra are similar in their spectral shape and are characterized by two resolved low- and high-energy components, Cα and Cβ, at the E_bind_’s of 284.8 eV and 286.2 eV (H_2_(Salen)), and 285.75 eV and 287.25 eV ([Ni(Salen)]), which are separated from each other by a close energy distance Δ, equal to 1.4 eV and 1.5 eV, respectively. In addition, the spectra show a low-intensity broad band *sh* over the E_bind_ range of 290–292 eV. It should also be noted that these results for the complex agree in terms of the spectral shape and energy positions of the PE bands with the data that were previously obtained using synchrotron radiation [33].

At the same time, the compared PE spectra reveal significant differences in the energy positions of individual components Cα and Cβ, their relative intensities, and their spectral resolution. Thus, when passing from the H_2_(Salen) spectrum to that of [Ni(Salen)], close high-energy shifts of 1.0 eV and 1.1 eV are observed for the Cα and Cβ PE components, respectively. The high-energy shift of the C 1s PE spectrum of [Ni(Salen)] as a whole, by about 1 eV relative to the H_2_(Salen) spectrum, unambiguously indicates a change in the chemical states of all the carbon atoms and an increase in their effective charges in the complex compared to the ligand molecule.

These energy shifts will be discussed below, simultaneously with consideration of the N 1s and O 1s PE spectra of H_2_(Salen) and [Ni(Salen)].

Along with this, there is a noticeable increase in the peak intensity of the Cβ band relative to the Cα one, and these bands also become better resolved. The fitting parameters for the compared spectra are presented, in detail, in Table S1. Obviously, the spectral shape of the C 1s PE spectra of H_2_(Salen) and [Ni(Salen)], consisting of two components, is due to the presence of two groups of carbon atoms in different chemical states in the atomic structure of the molecule and the complex (Figure 1). In a previous work [33], it was suggested that the carbon atoms linked to the oxygen and nitrogen atoms in [Ni(Salen)] are in close chemical states, which differ from those of the carbon atoms linked only to other carbon atoms. As a result, the sixteen carbon atoms of the [Ni(Salen)] complex were divided into two groups, Cα and Cβ: the first one consists of the C1 and C4 atoms of the phenyl rings of C_6_H_5_ (10 atoms in total) and the second one consists of the C2, C3, and C5 atoms (bonded to N or O atoms in addition to carbon atoms; 6 atoms in total). Such a division of the number of carbon atoms *n*(Cα): *n*(Cβ) = 1.0: 0.6 coincides practically with the peak intensity ratio of 1.0: 0.63 for the corresponding PE components in the [Ni(Salen)] spectrum (Figure 2a, Table S1).

In the H_2_(Salen) spectrum, the peak intensity of the Cβ band, with respect to that of the Cα band, is only 0.47 (Figure 1a), which is noticeably lower than the above-mentioned relative intensity of the Cβ band (0.63) in the [Ni(Salen)] spectrum. A comparison of the total intensities of the Cα and Cβ components, based on fitting the PE spectrum with two individual bands, only slightly increases the relative intensity of the Cβ component to a value of 0.52 (Table S1). Based on the relative intensity of the PE band Cβ, it can be assumed that the number of carbon atoms of Cβ and Cα in the H_2_(Salen) molecule are 5 and 11, respectively, since, in this case, the relative intensity of the Cβ component in the C 1s PE spectrum of H_2_(Salen) is expected to be 0.45, which is close to the observed intensity. It should be noted that this is a rather unexpected result, since the carbon atoms of Cβ in H_2_(Salen) (Figure 1) interact in pairs with oxygen atoms (two C5 atoms) and nitrogen atoms (two C3 atoms and two C2 atoms) and they should have the same chemical states in these pairs.

A significant redistribution of the charge (chemical) state of the carbon atoms in the salen ligand, in going from a molecule to a complex, is also evidenced by a comparison of the FWHM of the PE bands in the compared spectra. Indeed, the C 1s spectra of H_2_(Salen) and [Ni(Salen)] were measured under the same experimental conditions; nevertheless, the individual Cα and Cβ bands obtained as a result of fitting exhibit noticeably larger FWHM (1.09 and 1.25 eV, Table S1) in the spectrum of the molecule in comparison to the full widths of 0.97 eV for both bands in the spectrum of the complex. The Cα and Cβ bands in the PE spectrum of the H_2_(Salen) molecule are formed by PE signals from the 1s electron shells of the C1, C4 and C2, C3, C5 carbon atoms, respectively. Each type of carbon atom C1 … C5 is characterized by its own E_bind_ for the 1s electron shell and these energies differ little from each other within the groups of the Cα and Cβ atoms. However, this small spread in the binding energies of the C 1s electrons in a group leads to an additional broadening of the corresponding PE band beyond the instrumental FWHM. As can be seen in Figure 2a and Table S1, this broadening in the C 1s PE spectrum of the molecule is noticeably larger than in that of the complex.

It is now interesting to compare the binding energies of the C 1s electrons in the H_2_(Salen) molecule with the corresponding energies for the phenol and ethylenediamine fragments of this molecule. The energy position of the low-energy Cα component (284.80 eV) in the H_2_(Salen) spectrum agrees well with the E_bind_ of the 1s electrons of the carbon atoms in the phenol C_6_H_5_OH, which are not bound to the oxygen atom (284.5 eV [37]). In turn, the high-energy Cβ component is located at the E_bind_ of 286.2 eV, which coincides with the C 1s binding energy in the phenol or ethylenediamine C_2_H_4_(NH_2_)_2_ molecules for the carbon atoms that are bonded to oxygen or nitrogen atoms (286.1 eV [37] and 286.0 eV [38], respectively). Thus, the observed correlation between the energy positions of the Cα and Cβ components in the C 1s PE spectrum of the H_2_(Salen) molecule and the C 1s PE lines in the spectra of the phenol and ethylenediamine molecules confirms the correct assignment of the carbon atoms C1, C4 and C5, C2 to the two groups, Cα and Cβ, of the carbon atoms, which do not directly interact and interact with the oxygen and nitrogen atoms in H_2_(Salen). In this case, the decrease in the relative intensity of the Cβ band and the broadening of the Cα and Cβ bands in the H_2_(Salen) spectrum can be logically associated with the location of the C 1s PE line of the C3 atoms in the region of the binding energies between the Cβ and Cα bands.

Let us now compare the experimental C 1s PE spectra of the H_2_(Salen) molecule and the [Ni(Salen)] complex with their calculated spectra (Figure 2b). The calculated E_bind_ of the C 1s electrons in the different carbon atoms of the molecule and complex (Table S2) are shown as vertical black bars in Figure 2. Then, to facilitate this comparison with the experimental spectra, the obtained spectra (red curves) were aligned with the intensity of the Cα bands and shifted to the high-energy side by 5.64 eV (H_2_(Salen)) and 6.86 eV ([Ni(Salen)]), so that the Cα band coincided in its energy position with the corresponding bands in the experimental spectra. The main individual components (Cα, Cβ, Cβ_1_, and Cβ_2_) of the calculated spectra are shown by purple dashed lines.

As can be seen, the calculated spectra reproduce well the two-band shape of both spectra, the energy distance Δ between the Cα and Cβ bands, and the increase in the relative intensity of the Cβ band upon passing from the spectrum of the molecule to that of the complex. It is also important to note that the calculation confirms the assumption made above about a significant change in the 1s binding energies of the carbon atoms assigned to the Cβ group upon passing from the spectrum of [Ni(Salen)] to the spectrum of H_2_(Salen). Indeed, the 1s binding energies of the carbon atoms in H_2_(Salen) are divided into three groups (Cα, Cβ_2_, and Cβ_1_), while in the spectrum of [Ni(Salen)], a clear division into only two groups is observed.

The main difference between the compared spectra is that the relative intensity of the Cβ band in the calculated spectrum of H_2_(Salen) (0.33) is significantly less than the intensity in the experimental one (0.52), while these intensities practically coincide in the spectra of [Ni(Salen)] (Figure 2b, Table S1). Despite this discrepancy, we can state that the experimental and calculated results indicate a similar change in the chemical state of the carbon atoms in the salen ligand upon passing from a molecule to a complex. Indeed, the compared C1s PE spectra demonstrate a high-energy shift of about 1 eV, which indicates a decrease in the density of the valence electrons of the carbon atoms in the [Ni(Salen)] complex compared to those in H_2_(Salen). Obviously, this is a consequence of the chemical bonding between the complexing nickel atom and the oxygen and nitrogen atoms of the salen ligand.

**Figure 3:**
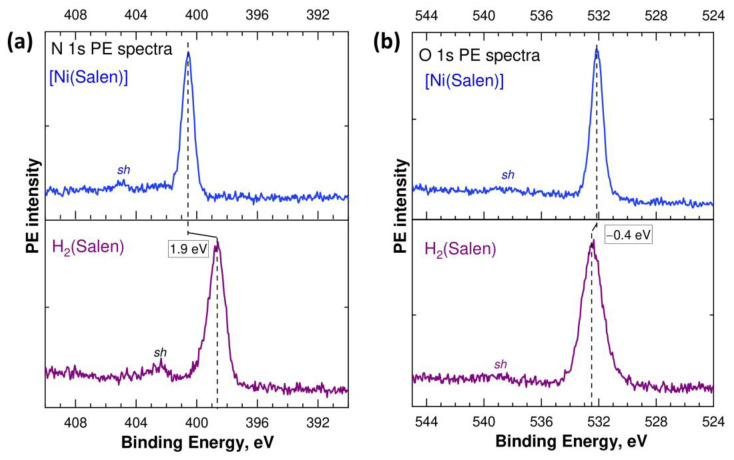
N 1s (**a**) and O 1s (**b**) PE spectra of the H_2_(Salen) molecule and [Ni(Salen)] complex measured using Al Kα radiation (*hν* = 1486.6 eV) and plotted on the scale of electron BE’s relative to the Fermi level.

In this regard, it is interesting to understand how the chemical states of oxygen and nitrogen atoms, which directly interact with the nickel atom during the formation of the complex, change. To do this, let us compare the measured N 1s and O 1s PE spectra of the salen ligand H_2_(Salen) and [Ni(Salen)] complex, which are presented in Figure 3 on the BE scale, relative to the Fermi level. The N 1s and O 1s PE spectra are represented by single lines at binding energies of 398.65 eV and 532.5 eV (H_2_(Salen)), and 400.55 eV and 532.10 eV ([Ni(Salen)]). The binding energies of the N 1s and O 1s electrons obtained for [Ni(Salen)] are in good agreement with the data (400.3 eV and 532.0 eV) of this work [33], in which these spectra were excited by monochromatic synchrotron radiation with photon energies of 500 eV and 615 eV, respectively.

The simple shape of the 1s PE spectra of the N and O atoms, in contrast to the 1s PE spectrum of the C atoms (Figure 2), implies that the chemical states of both nitrogen atoms (oxygen atoms) are the same in the ligand molecule and the complex. When comparing the binding energies of the N 1s and O 1s electrons in the molecule and complex, a significant high-energy shift of 1.9 eV is observed in the spectra of the nitrogen atoms and a small low-energy shift of –0.4 eV in the spectra of the oxygen atoms. Taking into account the positive charge of the Ni^2+^ cation and the high-energy shift found above for all the carbon atoms in the complex, it can be argued that the formation of the [Ni(Salen)] complex is accompanied by a significant redistribution of the electron density for all the atoms in the salen ligand, due to its transfer to the region of the oxygen atoms. In other words, this means that the transfer of electron density to the O atoms in [Ni(Salen)] occurs not only from the Ni atom, but also from the N and C atoms. An important role in this process is apparently played by the delocalized conjugated pi system of the 2p electron states of carbon atoms.

Finally, all the C, N, and O 1s PE spectra of the ligand molecule and complex contain low-intensity satellites *sh* at higher E_bind_’s (Figure 2a and Figure 3), which usually accompany the photoionization of the 1s electron shells of light atoms and arise due to the shake-up processes of valence electrons [39,40]. In the C 1s spectra of H_2_(Salen) and [Ni(Salen)], these satellites are very low-intensity and broad due to the more complex shape of the parent C 1s PE line compared to that of the N 1s and O 1s PE line, and their energy position is difficult to characterize correctly. The energy positions of the shake-up satellites in the N 1s and O 1s PE spectra are 402.5 eV and 404.9 eV, and 538.8 eV and 538.4 eV, for H_2_(Salen) and [Ni(Salen)], respectively. The energy distances between the main lines and their satellites are equal to 4.4 eV (N 1s PE spectra) and 6.3 eV (O 1s PE spectra). Since these satellites are due to the additional excitation of the valence electrons from upper MOs localized on the nitrogen (oxygen) atom to the lower, unoccupied MOs [19], these values can be used to estimate the energy separation between the MOs participating in the shake-up process. They can also be regarded as upper bounds for the energy separation between the highest occupied MO (HOMO) and the lowest unoccupied MO (LUMO) in H_2_(Salen) and [Ni(Salen)]. The differences in the corresponding energy separations in the N 1s and O 1s PE spectra can be explained by the effect of the core hole on the excitation process of valence electrons.

### 2.2. UV PES

**Figure 4:**
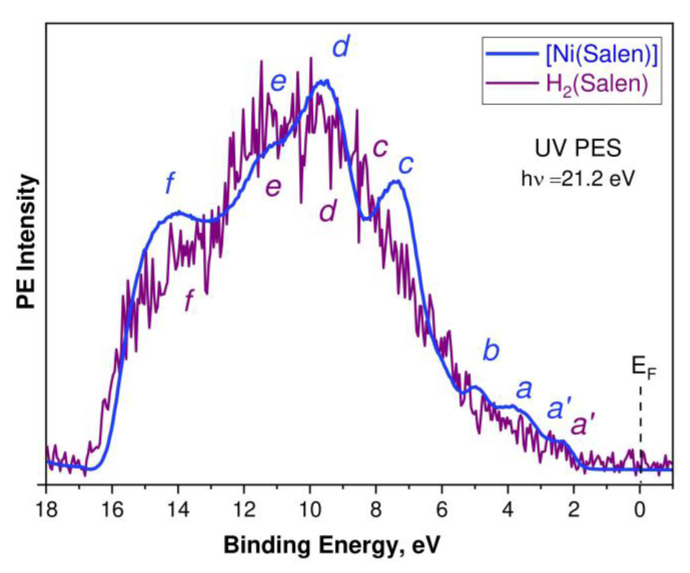
UV PE spectra of the H_2_(Salen) molecule (purple line) and the [Ni(Salen)] complex (blue line) excited by photon energies *hν* of 21.2 eV. Lowercase letters *a’*, *a*, *b*, *c*, *d*, *e*, and *f* designate the PE signals of individual subbands of the valence band. The spectra are normalized to the intensity of the PE band *d* and are plotted on the scale of valence electron BE’s relative to the Fermi level (*E*_F_).

Let us now consider the UV PE spectra of the H_2_(Salen) molecule and [Ni(Salen)] complex, which reflect the energy distribution and properties of the occupied electronic states in the valence band below the Fermi level. These spectra, measured at photon energies *hν* of 21.2 eV, are shown in Figure 4. They are normalized to the intensity of the PE band *d* and plotted on the scale of the valence electron BE’s relative to the Fermi level (*E*_F_). A preliminary discussion of these spectra was carried out in the framework of a study devoted to a consideration of the structural evolution of the VB PE spectra of the [Ni(Salen)] complex with a change in the energy of its exciting photons in the range of 21.2–848.0 eV [34]. Here, the qualitative results of this discussion are supplemented with a detailed comparative analysis of the UV PE spectra of H_2_(Salen) and [Ni(Salen)], using the results of the DFT calculations of their valence bands.

The H_2_(Salen) spectrum (Figure 4, purple line) is quite noisy due to sample charging; therefore, its structural elements are revealed only by a direct comparison with the [Ni(Salen)] spectrum (blue line). Both spectra are characterized by a similar spectral distribution of the PE intensities, with the most intense PE signals in the high E_bind_ range from 6 eV to 16 eV. This suggests that the energy distributions of the occupied electronic states in this energy region of the H_2_(Salen) and [Ni(Salen)] valence bands are very close to each other and are essentially determined by the corresponding states of the salen ligand. It should be noted here that this feature of the compared PE spectra is due to the dominance of the C 2p AOs of the phenol and ethylenediamine fragments in the formation of the electronic structure of the valence bands of the molecule and complex. Indeed, the number of carbon atoms in both compounds (16) is more than three times greater than the total number of oxygen, nitrogen, and nickel atoms (5). In addition, to characterize the PE spectrum, it is important that the photoionization cross-sections of the valence 2p AOs of the C, N, and O atoms and 3d AOs of the Ni atom are approximately equal (10–5 Mb) to an energy of the absorbed photons of 21.2 eV [41]. Therefore, the designations of the PE bands *c*–*f* in the H_2_(Salen) spectrum are made, taking into account their correlation with the corresponding PE bands in the [Ni(Salen)] spectrum and the results of their subsequent identification based on the DFT calculations.

The low-energy region of 2–6 eV in the [Ni(Salen)] spectrum is represented by the clear PE bands *a*′, *a*, and *b*, while it can be confidently assumed that the H_2_(Salen) spectrum contains only a weak *a*′ band at a binding energy of ~2.5 eV. At the same time, in the spectrum of H_2_(Salen), a structureless rise in the PE intensity is observed instead of the PE bands *a* and *b* in the [Ni(Salen)] spectrum. The PE bands *a* and *b*, located in the [Ni(Salen)] spectrum at the binding energies of 3.6 and 5.2 eV, were previously assigned to the photoionization of the valence MOs, with significant contributions from the valence 3d orbitals of the Ni atom [33]. Therefore, it can be assumed that the disappearance of bands *a* and *b* in the UV PE spectrum of the H_2_(Salen) molecule is associated with the absence of the Ni 3d-derived MOs in its valence band. In this case, it is logical to attribute the *a*′ band, as in the [Ni(Salen)] spectrum [33], to the photoionization of HOMO, formed by the π C2p MOs of the phenol fragments of the H_2_(Salen) molecule.

**Figure 5:**
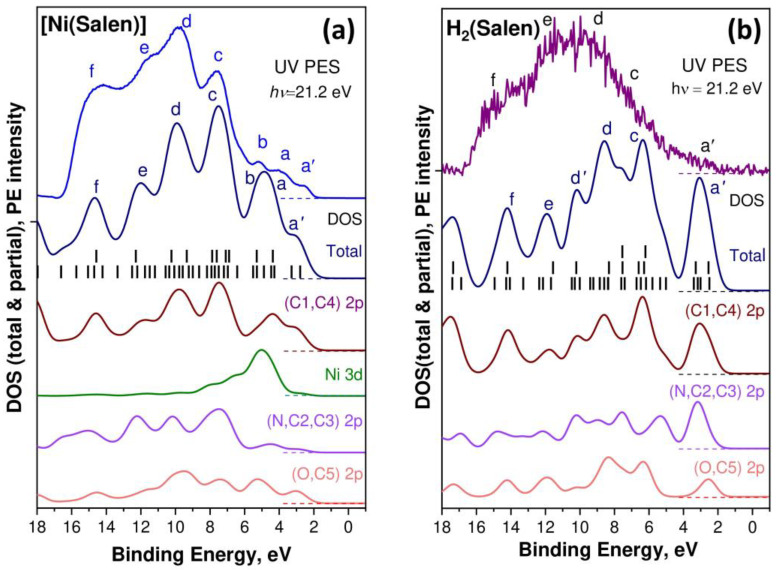
Comparison between UV PE spectra and valence band DOS based on DFT calculations for [Ni(Salen)] (**a**) and H_2_(Salen) (**b**). Calculated energy positions of valence MOs, shown by vertical black bars, were convoluted with a Gaussian contour with FWHM equal to 1.0 eV to facilitate the comparison with the experimental spectra. Lowercase letters *a’*, *a*, *b*, *c*, *d*, *e*, and *f* designate the PE signals of individual subbands of the valence band.

In order to more fully understand and describe the changes in the electronic structure of the salen ligand upon passing from the H_2_(Salen) molecule to the [Ni(Salen)] complex, DFT calculations of the valence bands for both compounds were performed. In Figure 5, the calculated spectra of the total and partial densities of the occupied valence states for [Ni(Salen)] (*a*) and H_2_(Salen) (*b*) are compared with the experimental UV PE spectra in the binding energy range from −1 to 18 eV. The corresponding spectra are aligned at the energy position of the *a*′ band. The higher occupied MOs of the H_2_(Salen) molecule and [Ni(Salen)] complex are shown in Figure S2.

First of all, it should be emphasized that the total DOS calculated for [Ni(Salen)] in this work does not practically differ from the data of the previous study [33]. The small differences in the intensities of the individual bands of the total and partial DOS of the occupied electronic states are apparently due to the use of a more powerful all-electron aug-cc-pVTZ basis in this work. When comparing the DOS spectra calculated for [Ni(Salen)] and H_2_(Salen), first of all, we note a significant difference in their total DOS spectra in the E_bind_ region below 4 eV, namely: a single band *a*′ in the spectrum of the H_2_(Salen) molecule is structured in the form of three bands *a*′, *a*, and *b* in the spectrum of the [Ni(Salen)] complex. In addition, the *d*′ and *d* bands in the total DOS spectrum for H_2_(Salen) merge into one band upon passing to the DOS spectrum of the complex. The partial DOSs of the ligand atoms are also characterized by significant changes in their spectral distributions upon passing from the molecule to the complex. Thus, in the case of [Ni(Salen)], in comparison with H_2_(Salen), the center of gravity of the partial N 2p and C 2p DOS is shifted to the region of a higher E_bind_, while that of the O 2p DOS is shifted in the opposite direction. This finding unambiguously indicates an increase in the effective charges of the carbon and nitrogen atoms, as well as a decrease in the effective charge of the oxygen atoms in the ligand during the formation of the [Ni(Salen)] complex. It should be emphasized that these directions of charge transfer are in perfect agreement with those established above when comparing the chemical shifts of the 1s PE spectra of the C, N, and O atoms for H_2_(Salen) and [Ni(Salen)].

As can be clearly seen from Figure 5, the spectra of the partial DOS for all the atoms strongly overlap in the entire valence band of the molecule and the complex, which reflects the strong mixing of the valence AOs due to the low symmetry of the compounds. This factor complicates the unambiguous assignment of the bands in the UV PE spectra to the valence MOs, with contributions from specific AOs. However, a careful examination of the partial DOS spectra allows us to assume that the PE band *a*′ in the UV PE spectra of both compounds is due to the photoionization of HOMO, formed mainly by the atomic 2pπ orbitals of the carbon atoms (C1 and C4) of the phenolic fragments. In turn, the atomic 2pσ orbitals of the C1 and C4 atoms are apparently responsible for the PE bands *c* and *d* in the spectra of the complex and the molecule. It is also logical to assert that the atomic N 2p and 2p orbitals of the C2 and C3 carbon atoms of the ethylenediamine fragment significantly contribute to the MOs responsible for the PE bands *c*–*e* in the [Ni(Salen)] spectrum. Finally, the results of the performed calculation confirm the important role of the Ni 3d states in the formation of the *a*–*d* bands of the occupied electronic states in the valence band of the [Ni(Salen)] complex, which was first pointed out in [33]. A more detailed analysis of the electronic structures of the valence bands for the H_2_(Salen) molecule and the [Ni(Salen)] complex requires additional calculations, taking into account the site symmetry for individual atoms.

### 2.3. NEXAFS

To obtain information about low-lying unoccupied electronic states in a polyatomic system, one should consider the NEXAFS spectra of the atoms forming this system. It is conventional to describe these spectra using the multiple (resonant) scattering of photoelectrons ejected from the core of an absorbing atom on its nearest neighbor atoms [17,18]. At certain photoelectron energies, a quasi-molecular fragment formed by neighborhood atoms can temporarily trap a photoelectron, resulting in the formation of metastable states (shape resonances). These resonances, depending on their lifetime, are observed in the spectra as narrow lines or broad absorption bands. Resonance localization in the quasi-molecule field allows us to consider them as a result of the dipole-allowed transitions of core electrons to the unoccupied MOs of this quasi-molecular fragment.

**Figure 6:**
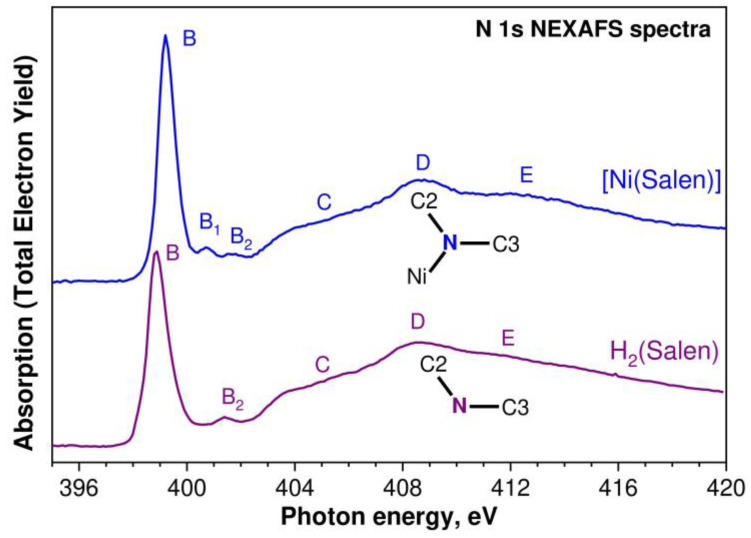
N 1s NEXAFS spectra of the H_2_(Salen) ligand (purple line) and the [Ni(Salen)] (blue line) complex. Capital letters *B*, *B_1_*, *B_2_*, *C*, *D*, and *E* denote X-ray absorption bands due to the N 1s electron transitions to unoccupied electronic states. The spectra are normalized to the same level of the N 1s continuous absorption at the photon energy of 420 eV. Inserts show the nearest atomic surroundings of absorbing nitrogen atoms in H_2_(Salen) and [Ni(Salen)] (from Figure 1).

Let us begin our comparative analysis of the obtained X-ray absorption spectra of the H_2_(Salen) molecule and [Ni(Salen)] molecular complex by considering the 1s NEXAFS spectra of the nitrogen and oxygen atoms. These atoms directly interact with the nickel cation in the complex; therefore, their spectra are expected to most fully reflect the changes in the local electronic structure of the salen ligand during the formation of the Ni complex. The N 1s NEXAFS spectra of the H_2_(Salen) ligand and [Ni(Salen)] complex are shown in Figure 6 as a dependence of the total electron yield (TEY) of the photoemission on the energy *hν* of the absorbed photons. The designations of the absorption structures in both spectra correspond to those of the structures for the [Ni(Salen)] complex spectrum in our previous work [33].

It is clearly seen that the compared spectra are characterized by a strong similarity in their overall spectral distribution in the photon energy range of 395–420 eV. In both spectra, the low-energy region is dominated by an intense absorption resonance *B* at the photon energies of 398.85 eV and 399.20 eV for H_2_(Salen) and [Ni(Salen)], respectively. In the H_2_(Salen) spectrum, this resonance is accompanied by a low-intensity band *B*_2_, an extended structureless rise *C*, and broad absorption bands *D*–*E* at the higher photon energies. These structures completely characterize the N 1s NEXAFS spectrum of H_2_(Salen). The main differences in the [Ni(Salen)] spectrum are a high-energy shift of the main absorption resonance *B* by 0.35 eV and a noticeable increase in its relative intensity, as well as the appearance of an additional low-intensity *B*_1_ band. These changes are observed in the low-energy region of the [Ni(Salen)] spectrum, in which absorption structures are associated with the transitions of the N 1s electrons to empty π*N 2p MOs [33]. All the other absorption bands, *C*–*E*, in the [Ni(Salen)] spectrum do not practically change their energy positions and relative intensities in comparison with those in the ligand spectrum.

It is well known that the 2p electronic states of the light atoms in planar molecules are split by the molecular field into the π2p_z_- and σ2p_x,y_-states, which characterize the occupied and vacant MOs of molecules oriented perpendicular and parallel to the molecular plane. Unoccupied electronic states are described by antibonding π* and σ* MOs, which appear in 1s NEXAFS spectra as π* and σ* absorption resonances due to dipole-allowed 1s→2p electronic transitions. The energy distance ΔE(π*–σ*) between them in the spectrum depends inversely on the distance between the absorbing atom and the atoms of its nearest surroundings [42]. In view of the above, it is logical to attribute the absorption resonances *D* and *E* in the N 1s NEXAFS spectrum of H_2_(Salen) to the N 1s electron transitions to the σ* MOs oriented to the carbon atoms C2 and C3, respectively, since the interatomic distances are *R*(N–C2) = 1.448 Å and *R*(N–C3) = 1.279 Å [35].

These absorption resonances, *D* and *E*, do not change their energy positions upon transitioning to the spectrum of [Ni(Salen)], which implies that the interatomic distances *R*(N–C2) and *R*(N–C3) in the complex remain unchanged. This conclusion agrees with the data for [Ni(Salen)] obtained via gas-phase electron diffraction [36].

The observed similarity of the N 1s NEXAFS spectra of the ligand molecule and the complex indicates weak changes in the local electronic and atomic structure of the salen ligand in the region of the nitrogen atom upon passing from H_2_(Salen) to [Ni(Salen)]. In other words, during such a transition, the spectrum of the vacant electronic states and the interatomic distances between the nitrogen atom and its nearest neighbors remain practically unchanged. In H_2_(Salen), the nitrogen atom is part of the imine (–C=N–) group linked with the phenolic and ethylene groups. In the [Ni(Salen)] complex, the nitrogen atoms are additionally coordinated by the complexing nickel atom, but this interaction has very little effect on the structure of the N 1s absorption spectrum, since the bonding is realized due to the lone pair of valence electrons of the nitrogen atom. Thus, this experimental finding for the N 1s NEXAFS spectra reflects the fact that the fine structure of the compared N 1s spectra is mainly determined by the electronic structure of the ethylenediamine bridge, while the interaction of the nitrogen and nickel atoms in the [Ni(Salen)] complex only leads to a change in the spectral parameters of the *B* resonance and the appearance of an additional low-intensity *B*_1_ structure.

As is known, the resonance *B* is due to the N 1s electron transitions to the unoccupied π*-states of the [Ni(Salen)] complex, which, in turn, can be described using the empty anti-bonding π*-MO of the NiN_2_O_2_ coordination center [33]. The existence of a similar absorption band in the Ni 2p_3/2_ and O 1s NEXAFS spectra of the coordination center indicates the presence of π bonding between the complexing Ni atom and the nitrogen and oxygen atoms of the ligand in [Ni(Salen)]. Hence, it is clear that the vacant π*-MO, which is responsible for the resonance *B*, is characterized by significant contributions from the valence AO atoms of the NiN_2_O_2_ quasimolecule (Ni 3d, N 2p, and O 2p). The formation of such a π-type MO in planar complexes is a signature of a π-back-donation between the nickel and ligand atoms [43]. It should be noted that the low-intensity absorption band *B*_1_ is apparently also associated with the transitions of the N 1s electrons to the vacant π*-MO, which reflects the presence of a weak π-conjugation between the nitrogen atom and the carbon atoms of the benzene ring (through the C3 carbon atom).

The high-energy shift of the band *B* in the N 1s NEXAFS spectrum of the complex is indicative of a higher positive effective charge on the N atom in [Ni(Salen)] compared to the salen ligand, which is consistent with the above results for the analysis of the N 1s PE spectra of the molecule and complex. Taking into account the π-back-donation effect, this means that the N 2p electron density shifts from the N atoms to the 3d AO of Ni and then to the 2p AO of the oxygen atoms. Since the N 2p population of the occupied valence π-MO decreases, the N 2p contribution to the unoccupied π*-MO increases. Thus, the transfer of the electron density (of the lone pair) of the nitrogen atom to the complexing nickel cation also explains the increase in the intensity of the absorption *B* resonance upon passing from the N 1s spectrum of the free ligand to the that of the complex.

**Figure 7:**
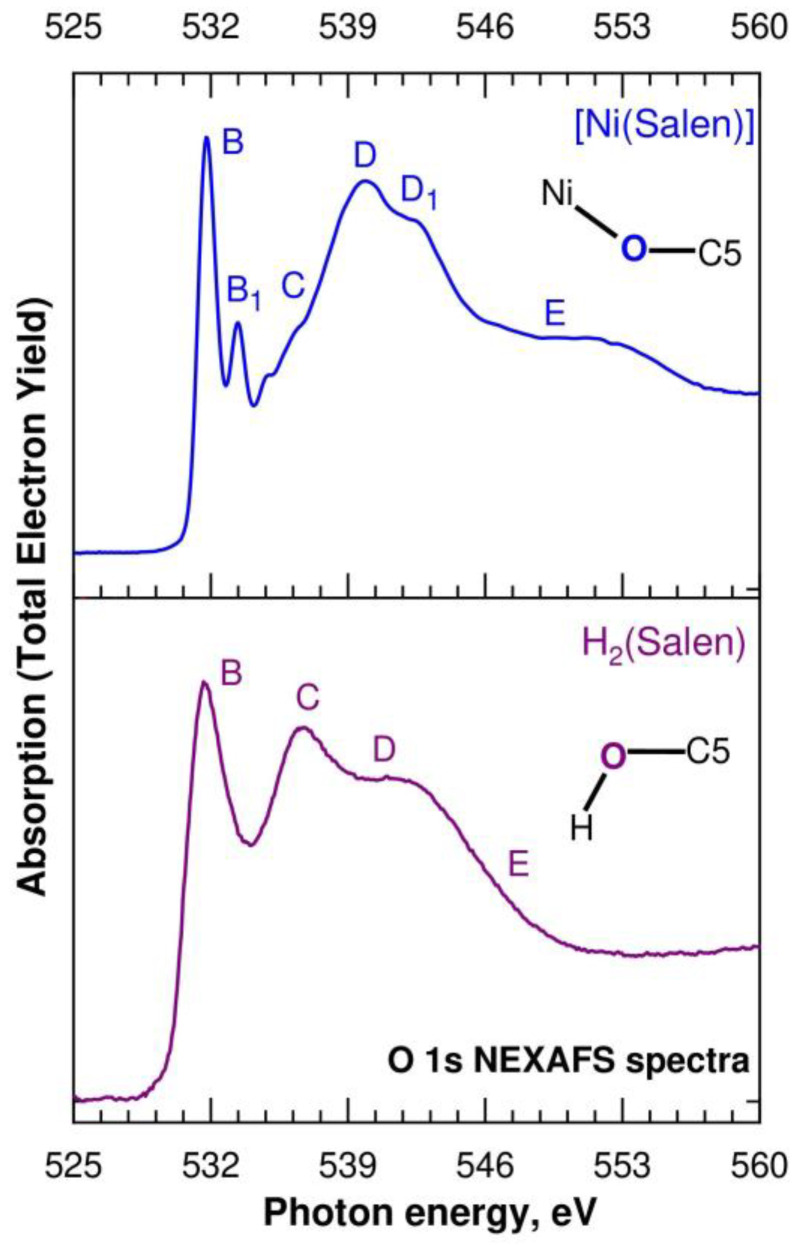
O 1s NEXAFS spectra of the H_2_(Salen) ligand (purple line) and the [Ni(Salen)] (blue line) complex. Capital letters *B*, *B_1_*, *C*, *D*, *D_1_*, and *E* denote X-ray absorption bands due to the O 1s electron transitions to unoccupied electronic states. The spectra are normalized to the same level of the O 1s continuous absorption at the photon energy of 560 eV. Inserts show the nearest atomic surroundings of absorbing oxygen atoms in H_2_(Salen) and [Ni(Salen)] (from Figure 1).

Let us now consider the O 1s NEXAFS spectra of H_2_(Salen) and [Ni(Salen)], which are shown in Figure 7 as a dependence of the TEY of the photoemission on the energy *hν* of the absorbed photons in the energy range from 525 eV to 560 eV. The designations of the absorption structures in both spectra correspond to those of the structures for the [Ni(Salen)] complex spectrum in our previous work [33]. The fine structure of the H_2_(Salen) spectrum consists of three broad absorption bands, *B*–*D*, and the last band *D* has an extended tail *E* on the high-energy side. These absorption structures correspond to the dipole-allowed transitions of the O 1s electrons to the unoccupied MOs localized in the region of the C–O–H molecular fragment, with significant contributions from the O 2p valence states. It is clearly seen that the fine structure of the O 1s absorption spectrum, in contrast to the N 1s spectrum, undergoes significant changes in going from H_2_(Salen) to [Ni(Salen)]. Thus, in the low-energy part of the spectrum of the complex, the absorption band *B* at a photon energy of 531.7 eV is split into two narrow absorption lines, *B* and *B*_1_ (531.8 eV and 533.4 eV), the relative intensities of the *C* and *D* bands are reversed, a distinct shoulder *D*_1_ appears near the *D* band. It is clearly seen that the fine structure of the O 1s absorption spectrum, in contrast to the N 1s spectrum, undergoes significant changes in going from H_2_(Salen) to [Ni(Salen)]. Thus, in the low-energy part of the spectrum of the complex, the absorption band *B* at a photon energy of 531.7 eV is split into two narrow absorption lines, *B* and *B*_1_ (531.8 eV and 533.4 eV), the relative intensities of the *C* and *D* bands are reversed, a distinct shoulder *D*_1_ appears near the *D* band, and finally, the high-energy extended tail *E* transforms into a broad band. The absence of narrow π-like absorption bands in the low-energy part of the H_2_(Salen) spectrum unambiguously indicates that the total molecular field in the region of the C–O–H fragment is very different from the flat one, due to its different orientations in the molecule. It is quite possible that this finding is partly due to the presence in the sample of several isomeric structures, in which these fragments have different orientations in the H_2_(Salen) molecule. As a consequence, the absorption transitions of the O 1s electrons to a vacant low-energy MO with O 2p contributions have slightly different energies, which explains the large width of the low-energy absorption band *B* in the H_2_(Salen) spectrum.

In moving from H_2_(Salen) to [Ni(Salen)], the salen ligand loses its hydrogen atoms and its oxygen atoms are linked to the complexing Ni atom. In the [Ni(Salen)] complex, the oxygen atoms, together with the nitrogen atoms, are coordinated by the Ni atom and form a square-planar coordination center, NiO_2_N_2_ [36]. This increase in the symmetry of the molecular field in the region of the oxygen atoms now makes it possible to consider the O 2p electronic states as split into 2p*π* and 2pσ components. Thus, it is logical to attribute the narrow absorption resonances *B* and *B*_1_ in the [Ni(Salen)] spectrum to the transitions of the O 1s electrons to two vacant antibonding π* MOs, separated by 1.6 eV from each other. The first one reflects the presence of π-bonding with the Ni atom (π-back-donation) [33], while the second π*-MO seems to indicate π-conjugation between the oxygen atoms and the benzene ring. It should be noted that a similar assignment of the absorption band *B*_1_ was made above when discussing the N 1s NEXAFS spectra of H_2_(Salen) and [Ni(Salen)]. It is important that the energy distance between the corresponding resonances *B* and *B*_1_ practically coincides in the spectra of the oxygen (1.6 eV) and nitrogen (1.5 eV) of the [Ni(Salen)] complex. The high relative intensity of the *B*_1_ resonance in the oxygen spectrum, in comparison with the nitrogen one, apparently indicates a higher degree of π-conjugation between the oxygen atoms and carbon atoms of benzene rings in the complex.

Using an analogy with the N 1s spectrum, it is constructive to attribute the absorption structures *C*–*E* in the O 1s spectrum of H_2_(Salen) to the transitions of the O 1s electrons to vacant antibonding σ*O 2p MOs, reflecting the σ-bonding of the O atom with the nearest hydrogen and carbon atoms. It can be seen from Figure 7 that, upon transitioning to the spectrum of [Ni(Salen)], the *C* band practically disappears, which makes it possible to associate it with the σ* MO, which is responsible for the σ bonding of the oxygen atom with the hydrogen atom. In this case, the *D* band can be attributed to σ* MO, which characterizes the chemical bonding between the oxygen and carbon (C5) atoms (Figure 1). It is important that this assignment is consistent with the presence of this absorption structure in the O 1s spectrum of [Ni(Salen)]. It is quite possible that the appearance of an additional absorption band *E* in the spectrum of the complex, which is absent in the spectrum of H_2_(Salen), is due to the σ bonding of the oxygen atom with the complexing Ni atom.

**Figure 8:**
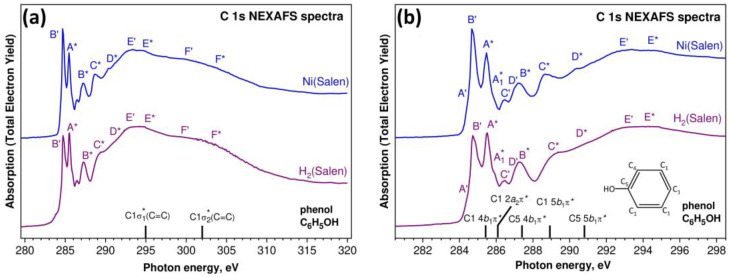
C 1s NEXAFS spectra of the H_2_(Salen) ligand and the [Ni(Salen)] complex (powders) in the photon energy range of 280–320 eV (**a**) and on the enlarged energy scale (281–298 eV) (**b**). Capital letters with dashes and asterisks denote X-ray absorption bands due to the transitions of C 1s electrons of ethylenediamine and phenolic groups to unoccupied electronic state, respectively. The spectra are normalized to the same level of the C 1s continuous absorption at the photon energy of 320 eV. The designations of carbon atoms in the phenol molecule (insert) are made taking into account the numbering in Figure 1. Vertical black bars with notations show the main transitions and their assignments in the C 1s absorption spectrum of the phenol molecule [37,44].

Let us now compare the C 1s NEXAFS spectra of the H_2_(Salen) ligand and the [Ni(Salen)] complex (Figure 8). Compared to the spectra of the nitrogen and oxygen atoms, the C 1s NEXAFS spectra show a richer fine structure, which is due to the presence of several chemically nonequivalent positions of the carbon atoms in the ligand (Figure 1). An examination of the spectra (Figure 8a) clearly shows that, as in the case of N 1s absorption spectra, the C 1s NEXAFS spectra of the free salen ligand and the [Ni(Salen)] complex are characterized by a strong similarity of their overall spectral distributions in the photon energy range of 280–320 eV and are consistent in the numbers of their main absorption bands and their energy positions. This finding is quite obvious, since the carbon atoms of the ligand do not directly interact with the complexing Ni atom. Nevertheless, when passing from a free ligand to a complex, small differences are still observed in the structure of the absorption spectrum of the [Ni(Salen)] complex, namely, (i) the relative intensity of the first absorption peak *B*’ noticeably increases, and (ii) the shoulders C* and D* become more contrasted and turn into clear absorption bands C* and D*. It is clear that these spectral differences are a consequence of the indirect influence of the nickel atom through the nitrogen and oxygen atoms on the properties of the vacant MOs spatially localized in the region of the carbon atoms. It is also possible that these changes are partly associated with an increase in molecular symmetry upon moving from H_2_(Salen) to [Ni(Salen)].

As noted above, carbon atoms are part of two polyatomic fragments (quasimolecules) of the ligand–phenolic and ethylenediamine groups. Let us begin a detailed discussion of the compared C 1s NEXAFS spectra by considering the contribution to the fine structure of the 1s excitations of the C atoms of the phenolic groups, since these C atoms predominate in the ligand. It is well known that the C 1s NEXAFS spectrum of the phenol molecule C_6_H_5_OH is very similar to that of the parent benzene molecule C_6_H_6_ [37,44]. At the same time, the addition of an electronegative oxygen atom to the benzene ring is accompanied by noticeable changes in the electronic structure of this benzene molecule, which are reflected in the C 1s NEXAFS spectrum of the phenol group.

Generally speaking, all the carbon atoms in a phenol molecule are in a different chemical (charge) states, since an electronegative oxygen atom attached to one of the C atoms of the benzene molecule C_6_H_6_ causes a redistribution of the electron density throughout the benzene ring. However, these differences are small for all the C atoms of the benzene ring, with the exception of the atom bonded to the oxygen atom, and do not lead to noticeable chemical shifts of the 1s levels of these carbon atoms. This allows us to consider the carbon atoms of the phenol group in two chemical states—the C1(C4) atoms bonded only to other carbon atoms and the C5 atom bonded also to an oxygen atom (insert in Figure 8b). The correctness of this separation of the carbon atoms in the phenol group is confirmed by the results of the photoemission measurements of the core C 1s spectra for the phenol molecule [45] and the [Ni(Salen)] complex, which demonstrate the two-component shape with close energy separations between these components—1.8 eV and 1.5 eV, respectively.

In addition to the splitting of the C 1s levels in the phenol molecule, the oxygen atom attached to the parent benzene molecule lowers the point symmetry of the molecule from the D_6h_ group (C_6_H_6_) to C_2v_ (C_6_H_5_OH), which, in turn, causes changes in the spectrum of the unoccupied MOs upon going from benzene to phenol. As a result, low-energy π* MOs of e_2u_ and b_2g_ symmetry types unoccupied in the benzene molecule [46] are transformed in the phenol molecule into the 4b_1_ + 2a_2_ and 5b_1_ π* MOs, respectively [37,44]. Thus, the splitting of the LUMO of π*e_2u_ symmetry into two 4b_1_ and 2a_2_ components and the presence of two types of chemically different carbon atoms are characteristic features of the electronic structure of the phenol molecule.

The C 1s NEXAFS spectrum of a gaseous phenol molecule C_6_H_5_OH (insert in Figure 8b) is well known and quite fully interpreted [37,44]. The results of this interpretation are shown in Figure 8b by vertical black bars with the C 1s absorption transition assignments. It is clearly seen that these C 1s → π* transitions are in good agreement in terms of their energy positions with the absorption bands marked by letters with asterisks in the spectra of H_2_(Salen) and [Ni(Salen)]. It should also be noted that the E* and F* absorption bands in the spectra of H_2_(Salen) and [Ni(Salen)] also agree well in terms of their energy positions with those of the σ_1_* and σ_2_* resonances at 295 eV and 302 eV in the spectrum of the phenol molecule (Figure 8a). This implies that the association of two phenol molecules with the ethylenediamine group in the H_2_(Salen) ligand, as well as the subsequent formation of the [Ni(Salen)] complex, have little effect on the energy spectrum of the unoccupied MOs of the phenol fragment in the compared polyatomic systems. The small changes observed in the C 1s NEXAFS spectrum of the complex are most likely associated with an increase in the symmetry of the polyatomic system in going from H_2_(Salen) to [Ni(Salen)].

It follows from the above that the other bands marked with dashed letters are due to the absorption transitions of the 1s electrons of the carbon atoms in the ethylenediamine group. This fragment also contains carbon atoms in two chemical states, which is due to their nearest surroundings in the C_2_H_4_ ethylene molecule (C2 atoms) and the –N=C– imine functional group (C3 atoms). First of all, this approach makes it possible to relate the intense sharp peak *B’* at the photon energy of 284.75 eV in the spectra of H_2_(Salen) and [Ni(Salen)] to the transitions of the C 1s electrons to the empty antibonding π* MO of the C_2_H_4_ molecule (284.7 eV) [47]. Moreover, the broad bands *E’* and *F’* in the C 1s NEXAFS spectra of H_2_(Salen) and [Ni(Salen)] (~293 eV and ~301 eV) should be considered to be as a result of the electronic transitions in the unoccupied σ_1_* and σ_2_* MOs of the C_2_H_4_ molecule (292.6 eV and 301 eV). Finally, in this case, it is logical to assume that the C 1s excitations of the carbon atoms in imine functional groups are responsible for the weak absorption bands *A’*, *C’*, and *D’* in the compared spectra. An increase in the intensity of the *B*’ resonance in the [Ni(Salen)] spectrum indicates a certain effect of the complexing Ni atom on the atomic orbital composition of the unoccupied π* MO of the ethylene fragment, which manifests itself in an increase in the contribution of the valence C 2p states to this MO.

## 3. Materials and Methods

The Salen-type compound *N*,*N*′-Ethylene-bis(salicylimine), H_2_(Salen), was synthesized using previously described procedures [48,49,50], via a condensation of salicylaldehyde and 1,2-ethylenediamine in ethanol under reflux for 3 h. After reaching room temperature, the mixture was cooled in a freezer, and the insoluble precipitate was filtered off and recrystallized from ethanol to produce a yellow polycrystalline solid. The molecular complex [Ni(Salen)] was synthesized in powder form using the standard procedure [48,49,50] by adding Ni(Ac)_2_ to the ligand in the ethanol. The obtained powder was purified via recrystallization from ethanol followed by drying under vacuum at 80 °C.

The H_2_(Salen) and [Ni(Salen)] samples for investigation were: (i) original powders rubbed ex situ into the scratched surface of a platinum (titanium) foil, and (ii) thin polycrystalline layers (with thicknesses in the range of 5–30 nm), which were prepared in situ via a thermal evaporation of the dehydrated powders from a tantalum (or quartz) crucible and deposited on clean polycrystalline Pt (Ti) foils. The crucible temperature during the evaporation was set at about 520–550 K. The deposition rate was controlled using a quartz microbalance for about 0.6 nm/min. According to mass spectrometry data [36], the vapor phase over solid [Ni(salen)] consists of monomer molecules. Similarly, the gas phase of H_2_(Salen) is mainly the vapor of the most energetically stable isomer [35]. All the samples were about 7 × 7 mm^2^ in size.

All the PE measurements for H_2_(Salen) and [Ni(Salen)] were performed at the Resource center, “Physical methods of surface investigation”, of the Saint Petersburg University Research park (St. Petersburg, Russia), using a Thermo Fisher Scientific ESCALAB 250Xi Photoelectron Spectrometer with a two-detector hemispherical electron analyzer. All the PE spectra were measured in the normal photoemission geometry and the angle-integrated mode, relative to the Fermi level of the sample. The overview and single-line PE spectra were recorded using monochromatized AlKα radiation (1486.6 eV) in the Constant Analyzer Energy (CAE) mode at pass energies of 50 eV and 10 eV, respectively. The purity and chemical compositions of the prepared samples were controlled by measuring the overview PE spectra. These spectra for the samples are given in the Supplementary Materials (see Figure S1). Under these conditions, the total energy resolution of the measured CL PE spectra was 800 meV. The binding energy (E_bind_) scale was calibrated against the Pt 4f_7/2_ PE peak (E_bind_ = 71.1 eV [19]), which was recorded from a pure platinum foil. The charging of the powdered samples of H_2_(Salen) and [Ni(Salen)] under X-ray irradiation was eliminated by a compensation system, using irradiation of the samples with a low-energy electron flux. As a result, the CL PE spectra of the powdered and evaporated samples did not practically differ from each other.

The photons of the He I resonance line (21.2 eV) were used to excite the ultraviolet VB PE (UV PE) spectra from the evaporated samples of H_2_(Salen) and [Ni(Salen)]. These PE spectra were obtained at an analyzer pass energy of 1.0 eV, which provided a total energy resolution of 360 meV. When recording the VB PE spectra, the system of electron–ion charge compensation was not used due to technical limitations; therefore, the spectrum of the H_2_(Salen) sample turned out to be noisier than that of the [Ni(Salen)] complex. All the PE measurements were carried out under an ultrahigh vacuum (10^−10^ mbar) at room temperature. The experimental data were processed using the Thermo Avantage ver. 5.9931 software package. A detailed CL PE peak analysis was performed by peak fitting using the Gaussian/Lorentzian product formula from the Casa XPS 2.3.16 software [51].

The NEXAFS spectra for the powdered and evaporated H_2_(Salen) and [Ni(Salen)] samples were measured with high-energy resolution using monochromatic synchrotron radiation and facilities of the Russian–German beamline at the BESSY II electron storage ring (Berlin, Germany) [52]. These spectra were obtained in the total electron yield (TEY) mode via detecting sample drain currents at a photon incident angle of 45°. The photon energy resolutions in the vicinity of the O 1s (~530 eV), N 1s (~400 eV), and C 1s (~280 eV) absorption edges were 150, 90, and 60 meV, respectively. All the NEXAFS spectra were normalized to the incident photon flux, which was monitored by recording the photocurrent from a clean gold mesh. The photon energy was calibrated by measuring the Pt 4f_7/2_ PE signals excited by radiation in the first and second diffraction orders and taking their difference. The measurements were carried out at room temperature and a base pressure of around 2∙10^−10^ mbar. No noticeable effects of charging or a deterioration of the H_2_(Salen) and [Ni(Salen)] samples irradiated by an intense synchrotron beam were observed. The NEXAFS spectra from the powdered and evaporated samples were recorded several times from different points on the sample and their structures usually showed a good reproducibility.

All the calculations for the atomic and electronic structures were performed for the isolated H_2_(Salen) and [Ni(Salen)] molecules using the DFT. The optimal geometries of the salen ligand and complex were optimized using a hybrid B3LYP functional [53,54] (Table S3). Pople’s 6-311+G** basis [55], used for the salen ligand, was combined with the lanl2tz+ basic set [55], suitable for the metal atoms. No symmetry constrains were applied during the optimization. The core levels (CLs) and valence MOs were calculated with a CAM-B3LYP [56] functional, which takes into account long-range adjustment, coupled with the all-electron aug-cc-pVTZ basis [57], which is commonly used for accurate calculations of metal–organic complexes [58,59]. We clearly identified the MOs corresponding to the 1s CLs of the C, N, and O atoms, because they possessed a very high overlap with the 1s basic functions, with negligibly low contributions from other functions. The BE of the 1s CLs were defined as the energies of the corresponding MOs. The DFT calculations for the valence band were performed with the GAMESS-US package [60] and visualized using the ChemCraft ver. 1.8 software [61]. To analyze the projected density of the states (DOS), all the carbon atoms were divided into five groups, as was performed in Ref. [33]. For all the plots of the core levels and DOSs of the valence MOs, the energy levels were broadened by Gaussian lines with a full width at a half-maximum (FWHM) of 1 eV.

## 4. Conclusions

A comparative study of the electronic structure of the salen ligand in the H_2_(Salen) molecule and the [Ni(Salen)] complex was performed using the experimental methods of XPS, UV PES, and NEXAFS spectroscopy, along with DFT calculations. The analysis of the experimental data was carried out within the framework of the approach considering the salen ligand as a combination of phenolic and ethylenediamine fragments. The measured spectra of H_2_(Salen) and [Ni(Salen)] were considered to obtain information about the changes in the local atomic–electronic structure of the salen molecule and the chemical state of its atoms when the salen was included as a ligand in the nickel complex, as well as the directions of the electron transfer between the complexing nickel atom and the ligand atoms.

Significant chemical shifts were observed in the 1s PE spectra of all the atoms of the salen ligand when passing from a molecule to a complex, indicating a significant redistribution of the valence electron density between the atoms and a noticeable change in the chemical states of all the atoms in the complex compared to the ligand molecule. Different signs of chemical shifts indicate the directions of the charge transfer from the nitrogen and carbon atoms to the oxygen atoms. An important role in this process is apparently played by the delocalized conjugated π system of the C 2p electron states of the ligand molecule.

The DFT calculations of the total and partial DOS for the valence bands of H_2_(Salen) and [Ni(Salen)] described well the spectral shape of the UV PE spectra of both compounds and confirmed their experimental identification. In the case of [Ni(Salen)], as compared to H_2_(Salen), the center of gravity of the partial N 2p and C 2p DOS was shifted to a higher E_bind_, while the O 2p DOS was shifted in the opposite direction. This fact confirmed an increase in the effective charges of the carbon and nitrogen atoms, as well as a decrease in the effective charge of the oxygen atoms in the ligand during the formation of the [Ni(Salen)] complex.

The N 1s and C 1s NEXAFS absorption spectra of the free salen ligand and the [Ni(Salen)] complex were characterized by a strong similarity of their overall spectral distributions over the entire photon energy range and coincided in the numbers of their main absorption bands and their energy positions. An additional low-intensity band observed in the N 1s NEXAFS spectrum of the complex 1.5 eV above the main π* resonance was associated with the presence of another vacant π*-MO, due to the weak π-conjugation between the nitrogen atoms of the ethylenediamine group and the carbon atoms of the phenol fragment. The O 1s NEXAFS spectrum, in contrast to the N 1s and C1s spectra, underwent significant changes upon passing from H_2_(Salen) to [Ni(Salen)]. Narrow low-energy absorption resonances in the [Ni(Salen)] spectrum were associated with the transitions of the O 1s electrons to two vacant anti-bonding π*-MOs, separated by 1.6 eV from each other. The first of them reflected the presence of π bonding with the Ni atom (π back donation) and the second π* MO, apparently, indicated π conjugation between the oxygen atoms and the phenolic rings.

## Data Availability

Not applicable.

## Supplementary Materials

The following supporting information can be downloaded at: https://www.mdpi.com/article/10.3390/ijms24129868/s1.
