# Supplementary material for: A Comparative XPS, UV PES, NEXAFS, and DFT Study of the Electronic Structure of the Salen Ligand in the H2(Salen) Molecule and the [Ni(Salen)] Complex
Source: Int J Mol Sci. 2023 Jun 7;24(12):9868. doi: 10.3390/ijms24129868 (PMC10298361; doi:10.3390/ijms24129868)
Supplement: Supplementary file 1 [file ijms-24-09868-s001.zip › ijms-2439997-supplementary.pdf]

International Journal of
Molecular Sciences

MDPI

Supplementary information

A Comparative XPS, UV PES, NEXAFS and DFT study of the electronic structure of the Salen ligand in the H_2(Salen) molecule and the [Ni(Salen)] complex

Petr M. Korusenko [1], Olga V. Petrova [1,2], Anatoliy A. Vereshchagin [3], Konstantin P. Katin [4], Oleg V. Levin [3], Sergey V. Nekipelov [2], Danil V. Sivkov [1,2], Victor N. Sivkov [2] and Alexander S. Vinogradov [1,*]

1 Department of Solid State Electronics, V.A. Fock Institute of Physics, St. Petersburg State University, 7/9 Universitetskaya nab., 199034, Saint Petersburg, Russia; teiou@mail.ru (O.V.P.); danjorno@yandex.ru (D.V.S.); p.korusenko@spbu.ru (P.M.K.)
2 Institute of Physics and Mathematics, Komi Science Centre, Ural Branch of the Russian Academy of Sciences, 167982, Syktyvkar, Russia; nekipelovsv@mail.ru (S.V.N.); sivkovvn@mail.ru (V.N.S.)
3 Department of Electrochemistry, Institute of Chemistry, St. Petersburg State University, 7/9 Universitetskaya nab., 199034, Saint Petersburg, Russia; anatoliy_ve@mail.ru (A.A.V.); o.levin@spbu.ru (O.V.L.)
4 Department of Condensed Matter Physics, National Research Nuclear University "MEPhI", Kashirskoe Sh. 31, 115409, Moscow, Russia
* Correspondence: asvinograd@yahoo.de

As can be seen from Figure S1, all presented survey spectra of the metal-free H_2(Salen) molecule and the [Ni(Salen)] complex do not show any peaks from substrate elements or contaminant inclusions. Therefore, it can be stated that the sample layers prepared by evaporation and deposition on the Pt foil are of high purity.

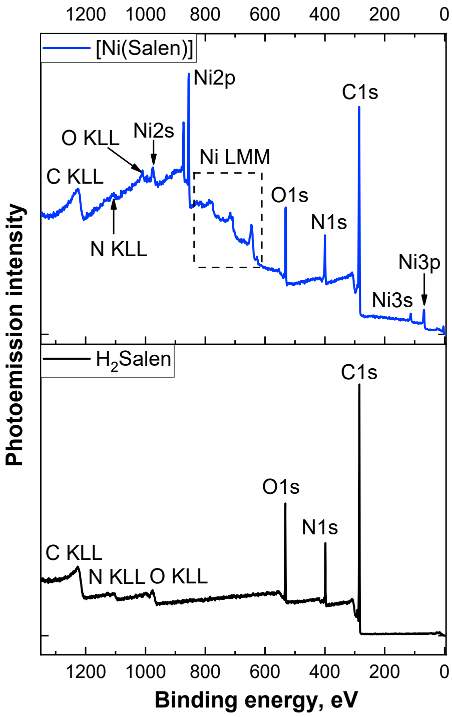

Figure S1. Overview PE spectra from the metal-free H_2Salen and [Ni(Salen)] complex deposited onto Pt foil were measured using ESCALAB 250 Xi electron spectrometer with Al Kα radiation ($h\nu$ = 1486.6 eV).

Table S1. Fitting parameters for C 1s PE spectra of H_2(Salen) and [Ni(Salen)].

Substance	PE band	E_{bind}, eV	FWHM, eV	Relative peak intensity	Relative total intensity
**Experiment**					
H_2(Salen)	Cα	284.78	1.09	1.0	1.0
	Cβ	286.16	1.25	0.47	0.52
[Ni(Salen)]	Cα	285.77	0.97	1.0	1.0
	Cβ	287.24	0.97	0.63	0.63
**Calculation**					
**Substance**	**PE band**	**E_{bind}, eV**	**FWHM, eV**	**Relative peak intensity**	**Relative total intensity**
H_2(Salen)	Cα	284.81	1.10	1.0	1.0
	Cβ1	285.59	1.0	0.165	0.165
	Cβ2	286.53	1.0	0.165	0.165
[Ni(Salen)]	Cα	285.78	0.97	1.0	1.0
	Cβ	287.49	0.97	0.60	0.60

Table S2. Calculated C 1s binding energies E_{bind} (in eV)* for different carbon atoms in the H_2(Salen) molecule and the [Ni(Salen)] complex.

Atom / Substance		C1 (8)	C2 (2) –N	C3 (2) –N	C4 (2)	C5 (2) –O
H_2(Salen)	E_{bind}	284.53—284.87	284.92	285.50	284.86	286.54
[Ni(Salen)]	E_{bind}	285.38—286.03	287.33	287.71	285.84	287.44

* The calculated C1s binding energies E_{bind} are indicated taking into account the energy shifts of 5.64 eV and 6.86 eV for H_2(Salen) and [Ni(Salen)], respectively, to ensure the energy matching of the Cα bands in the calculated and experimental C1s PE spectra.

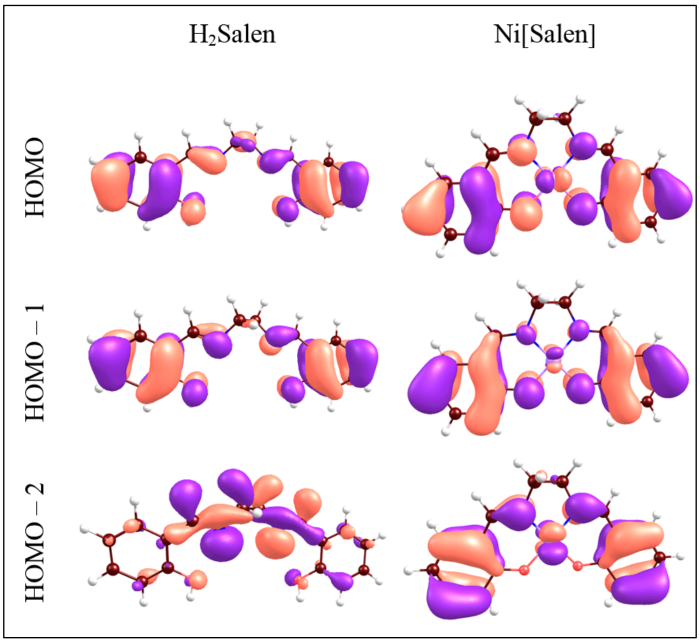

Figure S2. Higher occupied molecular orbitals of H_2(Salen) and [Ni(Salen)].

Table S3. Optimized Cartesian atomic coordinates (angstroms) of free Salen and [Ni(Salen)] complex.

H_2(Salen)				[Ni(Salen)]			
**Atom**	**x**	**y**	**z**	**Atom**	**x**	**y**	**z**
C	4.65588	−0.21927	−0.16965	Ni	1.1774	−2.03015	6.3E−4
C	−2.31653	−3.80039	0.1927	C	4.08562	−0.50511	0.15779
C	5.25018	−1.48792	0.01994	C	−1.75584	−3.50347	−0.15746
C	−1.64312	−5.02413	−0.02461	C	4.03458	−1.93944	0.13494
C	3.21225	0.03233	−0.30943	C	−0.56075	−4.29825	−0.13577
C	−1.66716	−2.49761	0.41145	C	2.89726	0.2611	0.01317
C	0.9276	−0.36181	−0.45957	C	−1.68541	−2.09141	−0.01001
C	−0.0025	−0.89483	0.64679	C	0.55957	0.67078	−0.33441
C	−3.72084	−3.82299	0.20277	C	−0.65548	0.04681	0.33857
C	5.50673	0.89671	−0.22374	C	−3.02257	−4.12641	−0.29615
C	−2.37846	−6.19789	−0.22188	C	5.33055	0.16107	0.29233
C	6.63941	−1.5937	0.14713	C	−0.71909	−5.71026	−0.25246
C	6.88671	0.79244	−0.09946	C	5.27472	−2.6337	0.24684
C	−4.45194	−4.98807	0.00642	C	6.50754	−0.54013	0.40646
C	7.45313	−0.46715	0.08824	C	−3.13836	−5.49113	−0.4158
C	−3.76921	−6.1841	−0.20778	C	6.46341	−1.95302	0.38039
N	−0.41952	−2.26329	0.40866	C	−1.96471	−6.27896	−0.39057
N	2.28946	−0.83916	−0.31404	N	−0.59333	−1.39548	0.07978
O	4.45636	−2.58622	0.0781	N	1.69521	−0.22074	−0.07603
O	−0.28658	−5.039	−0.03542	O	2.94679	−2.62321	0.01242
H	2.97234	1.10552	−0.41779	O	0.6289	−3.81314	−0.01065
H	−2.39115	−1.68166	0.58861	H	3.01213	1.34695	−0.03731
H	0.54388	−0.70901	−1.42554	H	−2.63464	−1.5521	0.04151
H	−0.86654	−0.2126	0.73439	H	0.39424	0.72249	−1.41685
H	0.87752	0.74128	−0.45501	H	−1.59006	0.48082	−0.03166
H	0.54429	−0.86414	1.59588	H	0.75182	1.68345	0.0351
H	−4.24284	−2.88612	0.37037	H	−0.60054	0.21281	1.42087
H	5.05555	1.87338	−0.36853	H	−3.91002	−3.49993	−0.30656
H	−1.84644	−7.13103	−0.38729	H	5.33842	1.24732	0.30308
H	7.08134	−2.57573	0.29379	H	0.18056	−6.31343	−0.23207
H	7.51026	1.67701	−0.14744	H	5.24024	−3.71628	0.22589
H	−5.53493	−4.96513	0.02035	H	7.45346	−0.0231	0.51094
H	8.52708	−0.57738	0.18943	H	−4.10964	−5.95798	−0.52396
H	−4.31445	−7.10836	−0.36332	H	7.38816	−2.51477	0.46697
H	0.01839	−5.93813	−0.20292	H	−2.04674	−7.35751	−0.48126
H	5.00499	−3.36619	0.22037				
